# Methodological Fallacies in the Determination of Serum/Plasma Glutathione Limit Its Translational Potential in Chronic Obstructive Pulmonary Disease

**DOI:** 10.3390/molecules26061572

**Published:** 2021-03-12

**Authors:** Salvatore Sotgia, Alessandro G. Fois, Panagiotis Paliogiannis, Ciriaco Carru, Arduino A. Mangoni, Angelo Zinellu

**Affiliations:** 1 Department of Biomedical Sciences, School of Medicine, University of Sassari, 07100 Sassari, Italy; panospaliogiannis@gmail.com (P.P.); carru@uniss.it (C.C.); azinellu@uniss.it (A.Z.); 2 Department of Clinical and Experimental Medicine, School of Medicine, University of Sassari, 07100 Sassari, Italy; agfois@uniss.it; 3 Department of Respiratory Diseases, University Hospital Sassari (AOU-SS), 07100 Sassari, Italy; 4 Discipline of Clinical Pharmacology, College of Medicine and Public Health, Flinders University and Flinders Medical Centre, Adelaide, SA 5042, Australia; arduino.mangoni@flinders.edu.au

**Keywords:** glutathione, low-molecular-weight thiols, COPD, systemic oxidation, redox state

## Abstract

This study aimed to review and critically appraise the current methodological issues undermining the suitability of the measurement of serum/plasma glutathione, both in the total and reduced form, as a measure of systemic oxidative stress in chronic obstructive pulmonary disease (COPD). Fourteen relevant articles published between 2001 and 2020, in 2003 subjects, 1111 COPD patients, and 892 controls, were reviewed. Nine studies, in 902 COPD patients and 660 controls, measured glutathione (GSH) in the reduced form (rGSH), while the remaining five, in 209 COPD patients and 232 controls, measured total GSH (tGSH). In the control group, tGSH ranged between 5.7 and 7.5 µmol/L, whilst in COPD patients, it ranged between 4.5 and 7.4 µmol/L. The mean tGSH was 6.6 ± 0.9 µmol/L in controls and 5.9 ± 1.4 µmol/L in patients. The concentrations of rGSH in the control group showed a wide range, between 0.47 and 415 µmol/L, and a mean value of 71.9 ± 143.1 µmol/L. Similarly, the concentrations of rGSH in COPD patients ranged between 0.49 and 279 µmol/L, with a mean value of 49.9 ± 95.9 µmol/L. Pooled tGSH concentrations were not significantly different between patients and controls (standard mean difference (SMD) = −1.92, 95% CI −1582 to 0.0219; *p* = 0.057). Depending on whether the mean concentrations of rGSH in controls were within the accepted normal range of 0.5–5.0 µmol/L, pooled rGSH concentrations showed either a significant (SMD = −3.8, 95% CI −2.266 to −0.709; *p* < 0.0001) or nonsignificant (SMD = −0.712, 95% CI −0.627 to 0.293; *p* = 0.48) difference. These results illustrate the existing and largely unaddressed methodological issues in the interpretation of the serum/plasma concentrations of tGSH and rGSH in COPD.

## 1. Introduction

Glutathione (GSH, l-*γ*-glutamyl-l-cysteinyl-glycine) is an intra- and extracellular reactive oxygen species (ROS) scavenger [1] that plays an essential role in maintaining the cellular redox state and in modulating immune and inflammatory responses [2,3,4]. These actions are particularly important in the lungs, where GSH is widely involved in protecting the membrane integrity of the airspace epithelium [5,6,7] from the toxic effects of several environmental sources of oxidants such as ozone, nitrogen dioxide, cigarette smoking, airborne pollution, particulates from car exhaust fumes, and occupational dust [8,9]. These noxious agents along with endogenous metabolic oxidants may increase the oxidative burden in the lungs and may trigger a significant local and systemic oxidative-driven inflammatory response underlying the development and progression of chronic obstructive pulmonary disease (COPD) [10,11,12]. Thus, it is not surprising that, in contrast with plasma or other bodily fluids, where the concentrations of GSH range between 2 and 4 µmol/L [13], the concentrations of GSH in the epithelial lining fluid (ELF), a thin aqueous continuous layer that covers the mucosa of the alveoli, the small airways, and the large airways, are up to 100-fold higher at 100–400 µmol/L [14]. In vivo and in vitro studies have reported a negative correlation between ELF GSH concentrations and the release of proinflammatory cytokines [15,16]. Moreover, a decrease in ELF GSH has been observed in other lung diseases such as idiopathic pulmonary fibrosis (IPF) [17], lung allograft [18], acute respiratory distress syndrome [19], and cystic fibrosis [20]. In COPD and asthma, ELF GSH is frequently found in a more oxidized state [21], further supporting the presence of a local pro-oxidant environment characterizing such conditions. Thus, ELF has been extensively collected for proteomic analyses and to detect the changes in the composition and concentrations of soluble components occurring in COPD or other lung diseases [22]. However, despite the potential utility of ELF for clinical and analytical purposes, the techniques required to harvest this biological matrix show some drawbacks, which greatly restrict its routine use. For example, ELF obtained from bronchoalveolar lavage fluid (BALF) is diluted by a factor of 60–120-fold, and there is no consensus on how to correct for this variation [23,24]. Similarly, the use of other biological matrices such as lung tissue, bronchial biopsies, induced sputum, exhaled breath condensate, and nasal lavage fluid, although useful to capture the local antioxidant/oxidant balance, raise similar issues in addition to being more or less invasive [22]. Conversely, blood matrices such as whole blood (WB), red blood cells (RBCs), and serum/plasma are relatively easy to collect and do not require specific preparation. In particular, blood GSH provides a simple measure of systemic oxidative stress and, indirectly, of the lung redox status. The fluctuation in blood GSH, in fact, may also affect its concentrations in ELF, as the amount of GSH in the latter may derive from circulating GSH originating from the liver [25]. However, as recently reported, the well-known methodological challenges linked to the assessment of GSH in WB and RBCs are rarely addressed [26]. While fallacies in the measurement of GSH in WB and/or RBCs may, to some extent, still provide useful information, flawed analyses using serum/plasma matrices may virtually abolish the translational potential of GSH measurements in COPD. This systematic review critically appraises the main methodological issues in studies investigating serum/plasma GSH concentrations in COPD and the extent to which they may limit the usefulness of this measurement.

## 2. Materials and Methods

### 2.1. Search Strategy and Studies Selection

A systematic electronic search of publications indexed in the PubMed and Web of Science databases, from inception until December 2020, was conducted using the following key words or their combinations: plasma/serum glutathione, GSH, chronic obstructive pulmonary disease, and COPD. Selection criteria included (i) original research study, (ii) assessment of GSH in serum or plasma, (iii) case–control design, (iv) clear description of analytical methods, (v) ≥10 patients with COPD, and (vi) full-text in English language. The references of the retrieved articles were also examined to identify potential additional studies. The articles were independently reviewed by two investigators and by a third in case of disagreement.

### 2.2. Statistical Analysis

Standard mean difference (SMD) was used to pool continuous data. Heterogeneity across studies was assessed using Cochran’s Q statistic, with a *p*-value of <0.1. The I^2^ statistic was used to measure the magnitude of heterogeneity. For I^2^ < 25%, heterogeneity was considered absent; for I^2^ between 25 and 50%, it was considered moderate; for I^2^ between 50 and 75%, it was considered large; and for I^2^ > 75%, it was considered extreme. A random-effect model was used to summarize the results in case of substantial heterogeneity. Tests were two-tailed, and a *p*-value <0.05 was considered statistically significant. The study was fully compliant with the principles outlined in the PRISMA Statement [27]. Statistical analyses were performed using MedCalc Statistical Software for Windows, version 17.5.5, 64 bit (MedCalc Software bvba, Ostend, Belgium).

## 3. Results

**Figure 1:**
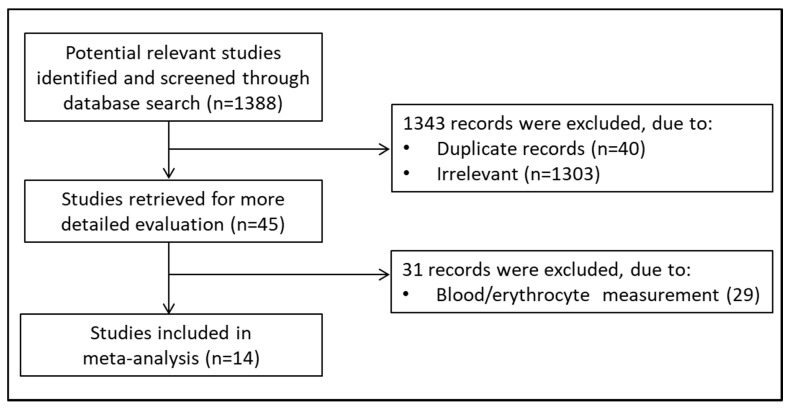
Flowchart of study selection.

**Figure 2:**
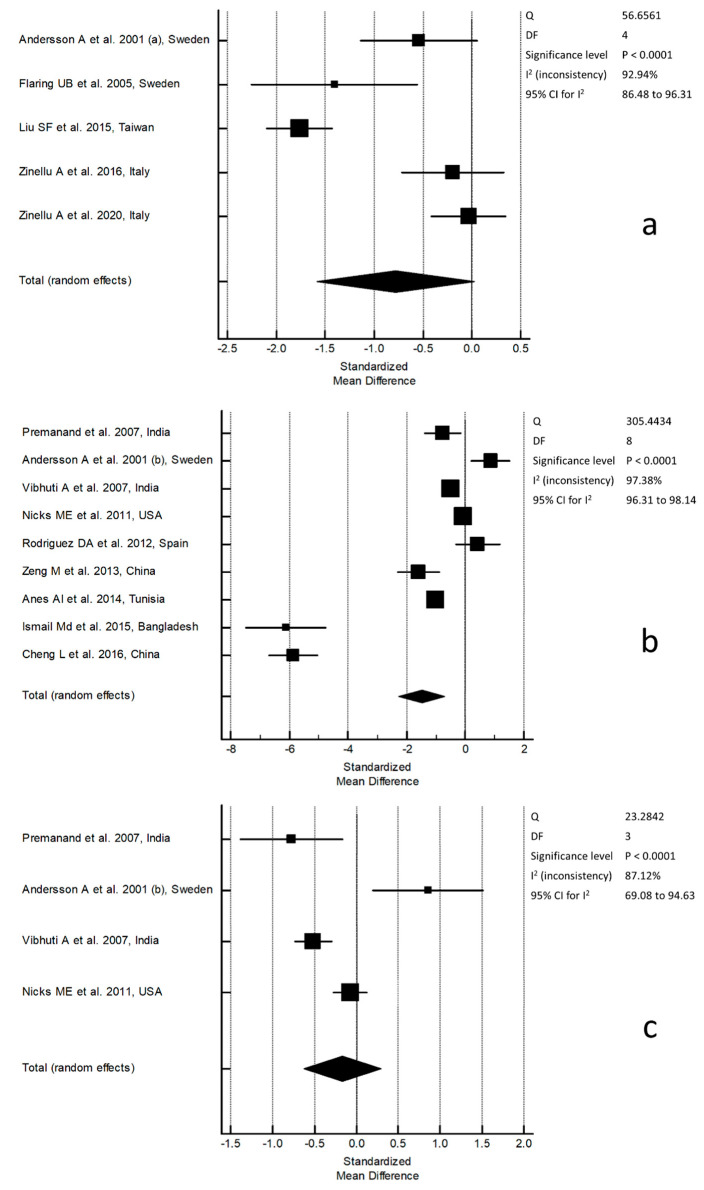
Forest plot of studies examining (**a**) serum/plasma total glutathione (tGSH) concentrations, (**b**) serum/plasma reduced glutathione (rGSH) concentrations, and (**c**) serum/plasma rGSH concentrations depending on whether rGSH fall into a cutoff for control groups of 0.5–5.0 µmol/L.

A flowchart describing the screening process is presented in Figure 1. The initial search yielded 1388 articles. Of them, 1343 were excluded because they were either irrelevant or duplicates. After a full-text review of the remaining 45 articles, a further 31 were excluded as they did not meet the inclusion criteria, leaving the remaining 14 for further analysis [28,29,30,31,32,33,34,35,36,37,38,39,40]. The retrieved studies were published between 2001 and 2020, in 2003 subjects, 1111 COPD patients, and 892 controls (Table 1). Nine studies, in 902 COPD patients and 660 controls, measured GSH in the reduced form (rGSH), while the remaining five, in 209 COPD patients and 232 controls, measured total GSH (tGSH). In three studies, GSH analysis was based on high-performance liquid chromatography (HPLC); two studies used capillary electrophoresis (CE); seven were based on spectrophotometric methods (Spectr); and two were based on two different commercial kits. Regardless of the method used, to improve the limit of detection, GSH was functionalized using derivatizing reagents. Ellman’s reagent (DTNB, 5,5′-dithiobis-(2-nitrobenzoic acid) was the most used (seven articles) followed by 2,2′-dithiodipyridine (two), 5-iodoacetamidofluorescein (5-IAF) (two), monobromobimane (one), and 1-methyl-2-vinylpyridinium trifluoromethanesulfonate (M2VP) (one). The concentrations of rGSH in the control group showed a wide range, between 0.47 and 415 µmol/L, and a mean value of 71.9 ± 143.1 µmol/L. Similarly, the concentrations of rGSH in COPD patients ranged between 0.49 and 279 µmol/L, with a mean value of 49.9 ± 95.9 µmol/L. In both controls and COPD patients, the concentrations of tGSH were within a narrower range than rGSH. In the control group, tGSH ranged between 5.7 and 7.5 µmol/L, whilst in COPD patients, they ranged between 4.5 and 7.4 µmol/L. The mean tGSH value was 6.6 ± 0.9 µmol/L in controls and 5.9 ± 1.4 µmol/L in patients. Extreme heterogeneity was observed in studies measuring tGSH (I^2^ = 92.94%, *p* < 0.0001). As shown in Figure 2a, the SMDs of tGSH concentrations in the COPD group were lower than the controls in two studies and with no between-group differences in three. As also shown in Figure 2a, pooled SMDs for tGSH (SMD = −1.92, 95% CI −1.582 to 0.0219; *p* = 0.057), assessed by a random-effect model, indicated the presence of a nonsignificant difference in concentrations between controls and COPD patients. By contrast, as shown in Figure 2b, the SMDs of rGSH concentrations were lower in the COPD group than in controls in six studies, higher in one, and with no between-group differences in two. Extreme heterogeneity was observed among studies (I^2^ = 97.38%, *p* < 0.0001), and pooled SMDs for rGSH (SMD = −3.8, 95% CI −2.266 to −0.709; *p* < 0.0001) assessed by a random-effect model indicated a significant difference in concentrations between controls and COPD patients. When considering those studies (*n* = 4) reporting, in control groups, a mean rGSH concentration between 0.5–5.0 µmol/L, the heterogeneity decreased (I^2^ = 87.12%, *p* < 0.0001); however the pooled SMD, computed by a random-effect model, was no longer significant (SMD = −0.712, 95% CI −0.627 to 0.293; *p* = 0.48) (Figure 2c).

## 4. Discussion

A recent meta-analysis showed that methodological factors may underlie the high variability observed in studies assessing GSH concentrations in whole blood (WB) and red blood cells (RBCs) as a measure of systemic oxidative state in COPD [26,41,42,43]. Unlike WB and RBCs, where GSH is found at millimolar levels, its concentrations in serum/plasma are in the micromolar range [13]. Therefore, methodological issues in the latter biological matrices may severely affect the accuracy of assessment, resulting in misleading measures of the different forms of GSH that ultimately curtail their interpretation. In intra- and extra-cellular environments, in fact, GSH may be found as disulfide and mixed-disulfides, referred to as oxidized-free forms (oxGSH), as protein-bound fraction (pGSH), and as reduced GSH (rGSH). Combined, they constitute the total fraction of GSH (tGSH), while oxGSH along with rGSH represents the free fraction of GSH (fGSH) [44]. Each fraction shows different concentrations that require specific analytical procedures [42,44,45]. On average, in healthy subjects, the reported plasma concentrations for each GSH’s form are 8.0 ± 1.4 µmol/L for tGSH, 3.5 ± 0.4 µmol/L for rGSH, 1.9 ± 0.4 µmol/L for oxGSH, 2.6 ± 0.8 µmol/L for pGSH, and 5.5 ± 0.7 µmol/L for fGSH (the sum of rGSH and oxGSH) [45]. However, redox conditions change rapidly after blood collection and any pre-analytical and analytical shortcomings may cause a significant shift in the ratios between different forms. Characterizing the usefulness of tGSH measurement in COPD in our systematic review was difficult due to the relatively small number of retrieved studies and the heterogeneity in methods used. To assess tGSH, in fact, a pre-analytical step where serum/plasma is treated with a reducing agent is mandatory. However, this key step was performed only in some studies, while in others, it was either not performed or done only after protein precipitation. Without the reduction step, the measured fraction does not represent tGSH but, rather, rGSH. In turn, the delayed reduction, after protein precipitation, does not allow for measuring tGSH but only fGSH as pGSH is lost with the removal of proteins. These discrepancies may explain the extreme heterogeneity observed between studies (I^2^ = 92.94%, *p* < 0.0001) and the slightly lower concentrations of tGSH observed in the control groups (6.6 ± 0.9 µmol/L) compared to the normally accepted values for healthy people (8.0 ± 1.4 µmol/L). Subject to these limitations, the pooled analysis using a random-effect model showed that tGSH concentrations were not significantly different in COPD patient vs. controls (Figure 2a). Unlike tGSH, a preliminary treatment with a reductive agent is not required in the assessment of rGSH. However, to avoid extracellular in vitro GSH autooxidation and a shift in redox state toward an artifact oxidative condition, blood should be drawn quickly under low temperatures [46] and serum/plasma should be separated from RBCs on a refrigerated centrifuge using a short centrifugation time [45]. Moreover, to prevent the change in redox state following blood collection, the prompt addition of reagents able to protect the free –SH group has also been suggested [47]. None of the identified studies adopted this strategy and only in some were the tubes placed in ice immediately after blood withdrawal. Although this might have contributed to inaccuracies in rGSH measurement, other factors are likely to account for the wide range of rGSH concentrations reported in the selected studies, 0.47–415 µmol/L with a mean value of 71.9 ± 143.1 µmol/L in the control groups and 0.49–279 µmol/L with a mean value of 49.9 ± 95.9 µmol/L in COPD patients. The review of these studies revealed that rGSH was mainly determined by spectrophotometric methods after derivatization with the Ellman’s reagent (DTNB, 5,5′-dithiobis(2-nitrobenzoic acid)). This is a powerful derivatizing reagent showing specificity for sulfhydryl compounds such as low-molecular-weight thiols [48]. As a result, it couples with amino thiols and sulfhydryl residues present in the sample rather than specifically to GSH [49]. Therefore, in the absence of HPLC or CE separation, technologies widely used in analytical laboratories [50,51,52,53,54], the spectrophotometric analysis alone is unable to discriminate between rGSH and several other potential derivatized thiols. The low-molecular-weight thiols that are functionally and/or metabolically close to GSH, such as homocysteine (Hcy), cysteinylglycine (CysGly), glutamylcysteine (GlyCys), and cysteine (Cys), might account for about 20 µmol/L of the overall concentrations measured in plasma and erroneously attributed to rGSH alone [45]. Thus, referring to this as a measure of rGSH is misleading as it does not target specifically GSH but, rather, each compound with a free –SH moiety in serum/plasma. This might account for the extreme heterogeneity observed between studies (97.38%, *p* < 0.0001), which, using a random-effect model, overall shows that rGSH concentrations are significantly different in COPD patients vs. controls (Figure 2b). However, establishing a concentration cutoff for control groups of 0.5–5.0 µmol/L based on the normally accepted values of rGSH [45] reduced the number of studies to four. Heterogeneity was greatly reduced (87.12%, *p* < 0.0001) and the pooled results showed that rGSH concentrations were not significantly different in COPD patients vs. controls (Figure 2c). Similar to WB and RBCs, but with a much greater impact, these conflicting results confirm the negative effect of these largely unaddressed methodological issues in the interpretation of the serum/plasma concentrations of tGSH and rGSH, therefore limiting the clinical applications of the assay in COPD. Nonetheless, it is worth noting that the measure of free –SH groups in serum/plasma, which includes also rGSH, is not without interest, provided that it is framed in the proper context. The greater concentration of free –SH groups in controls vs. patients, in fact, would indicate a more oxidative environment in the latter. This is consistent with a previous observation of a lower concentration of free –SH groups in plasma proteins of COPD patients than controls with a trend towards lower concentrations with increasing severity of the disease [31,32,55]. Therefore, a comprehensive redox state analysis of –SH groups rather than GSH alone might be particularly useful in the evaluation of systemic oxidative stress in COPD.

## Figures and Tables

**Table 1:** Summary of the studies on chronic obstructive pulmonary disease (COPD) vs. controls included in the study.

					Control Group		COPD Group	
First Author, Year, and Country	Assay Type	Derivatization Reagent	GSH Form	Measure Units	*n*	GSH Mean ± SD	*n*	GSH Mean ± SD
Andersson A et al., 2001, Sweden [28]	HPLC	dithiopyridine	t	μmol/L	29	5.69 ± 1.33	19	4.93 ± 1.43
Flaring UB et al., 2005, Sweden [29]	HPLC	monobromobimane	t	μmol/L	10	5.9 ± 1.03	21	4.6 ± 0.95
Liu SF et al., 2015, Taiwan [30]	Spectr	DTNB	t	mU/mL	110	6.8 ± 1.3	86	4.5 ± 1.3
Zinellu A et al., 2016, Italy [31]	CE	5-IAF	t	μmol/L	29	7.2 ± 2.6	29	6.7 ± 2.4
Zinellu A et al., 2020, Italy [32]	CE	5-IAF	t	μmol/L	54	7.5 ± 2.9	54	7.4 ± 3.0
Premanand et al., 2007, India [33]	Spectr	DTNB	r	μmol/ mg of protein	20	4.85 ± 0.97	20	5.72 ± 1.02
Andersson A et al., 2001, Sweden [28]	HPLC	dithiopyridine	r	μmol/L	29	1.58 ± 0.53	19	1.23 ± 0.26
Vibhuti A et al., 2007, India [34]	Spectr	DTNB	r	μmol/L	136	4.10 ± 2.64	202	3.06 ± 1.45
Nicks ME et al., 2011, USA [35]	Spectr	M2VP	r	μmol/L	136	0.53 ± 0.34	367	0.50 ± 0.42
Rodriguez DA et al., 2012, Spain [36]	Kit	NR	r	μmol/L	12	0.47 ± 0.04	18	0.49 ± 0.05
Zeng M et al., 2013, China [37]	Kit	NR	r	mg/L (μmol/L)	14	413 ± 8 ^#^ (1.340 ± 30)	35	352 ± 44 ^#^ (1.147 ± 143)
Ben Anes AI et al., 2014, Tunisia [38]	Spectr	DTNB	r	μmol/L	229	96 ± 58	153	47 ± 26
Ismail Md et al., 2015, Bangladesh [39]	Spectr	DTNB	r	μmol/L	20	415 ± 20	30	279 ± 23
Cheng L et al., 2016, China [40]	Spectr	DTNB	r	mg/L (μmol/L)	64	17.2 ± 1.2 ^#^ (56 ± 4)	58	10.6 ± 0.8 ^#^ (35 ± 3)

5-IAF: 5-iodoacetamidofluorescein; CE: capillary electrophoresis; M2VP: 1-methyl-2-vinylpyridinium trifluoromethanesulfonate; NR: not reported; ^#^ mg/L.

## Data Availability

Not applicable.
